# Supplementary material for: Cholecystokinin-A signaling regulates automaticity of pacemaker cardiomyocytes
Source: Front Physiol. 2023 Dec 21;14:1284673. doi: 10.3389/fphys.2023.1284673 (PMC10764621; doi:10.3389/fphys.2023.1284673)
Supplement: Supplementary file 1 [file DataSheet1.pdf]

SUPPLEMENTARY MATERIAL

Cholecystokinin-A signaling regulates automaticity of pacemaker cardiomyocytes

Hongmei Ruan*, Ravi Mandla, Namita Ravi, Giselle Galang, Amanda W. Soe, Jeffrey E Olgin, Di Lang, and Vasanth Vedantham*

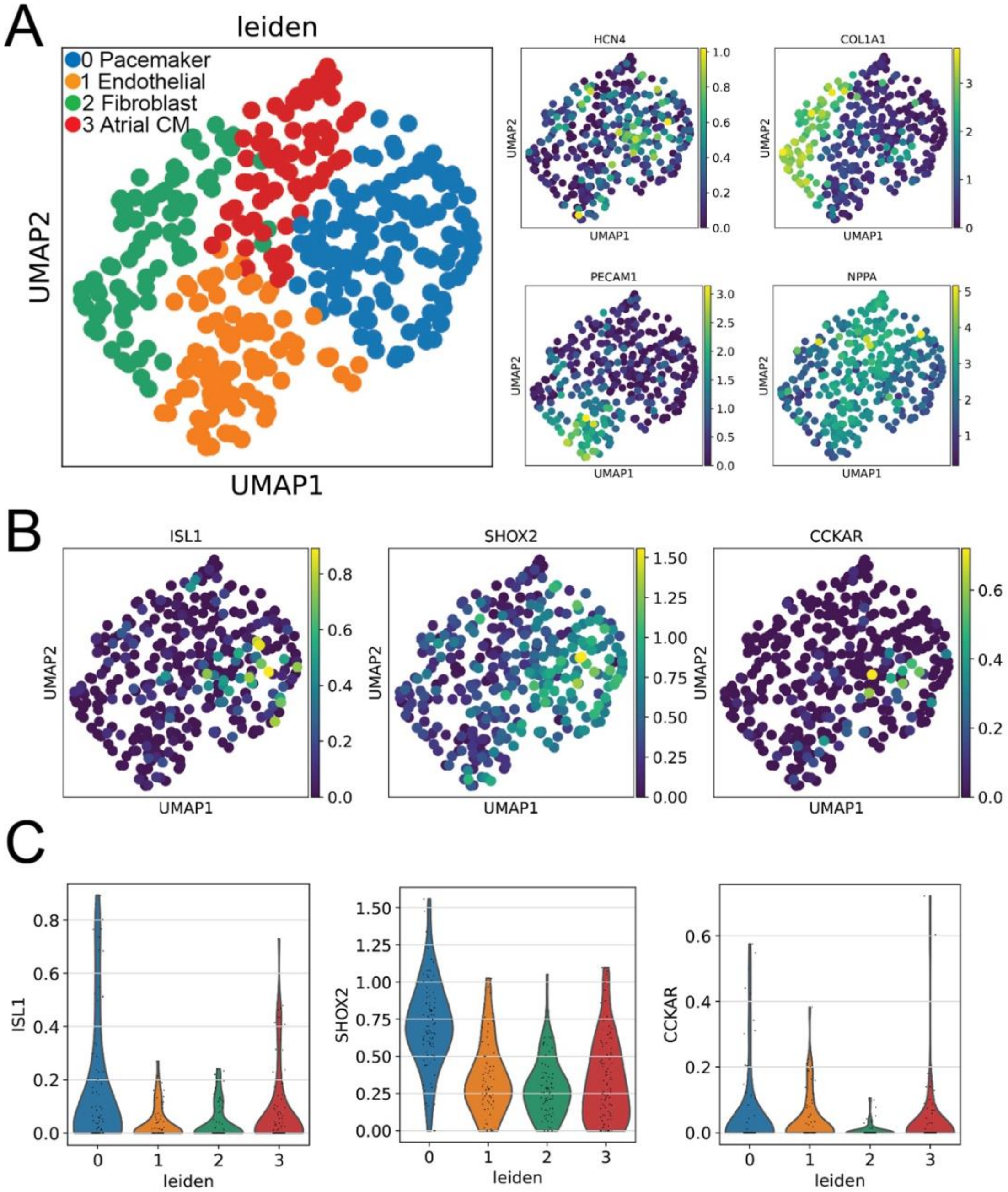

<u>Supplementary Figure 1. Expression of Cckar in Rabbit Sinoatrial Node</u>. (A). Uniform Manifold Approximation Projection (UMAP) plot of leiden clustering analysis of single cell RNA sequencing data derived from rabbit sinoatrial node tissue[1] demonstrated an *Hcn4*$^+$ pacemaker cardiomyocyte (PC) population (cluster 0), as well as *Pecam*$^+$ endothelial cells (cluster 1), *Col1a1*$^+$ fibroblasts (cluster 2), and *Nppa*$^+$ atrial cardiomyocytes (cluster 3) (B) UMAP plots demonstrating co-expression of established PC enriched genes *Shox2*, and *Isl1* alongside *Cckar* in cluster 0. (C) Quantification of expression of pacemaker genes Isl1, Shox2, and Cckar in different clusters.

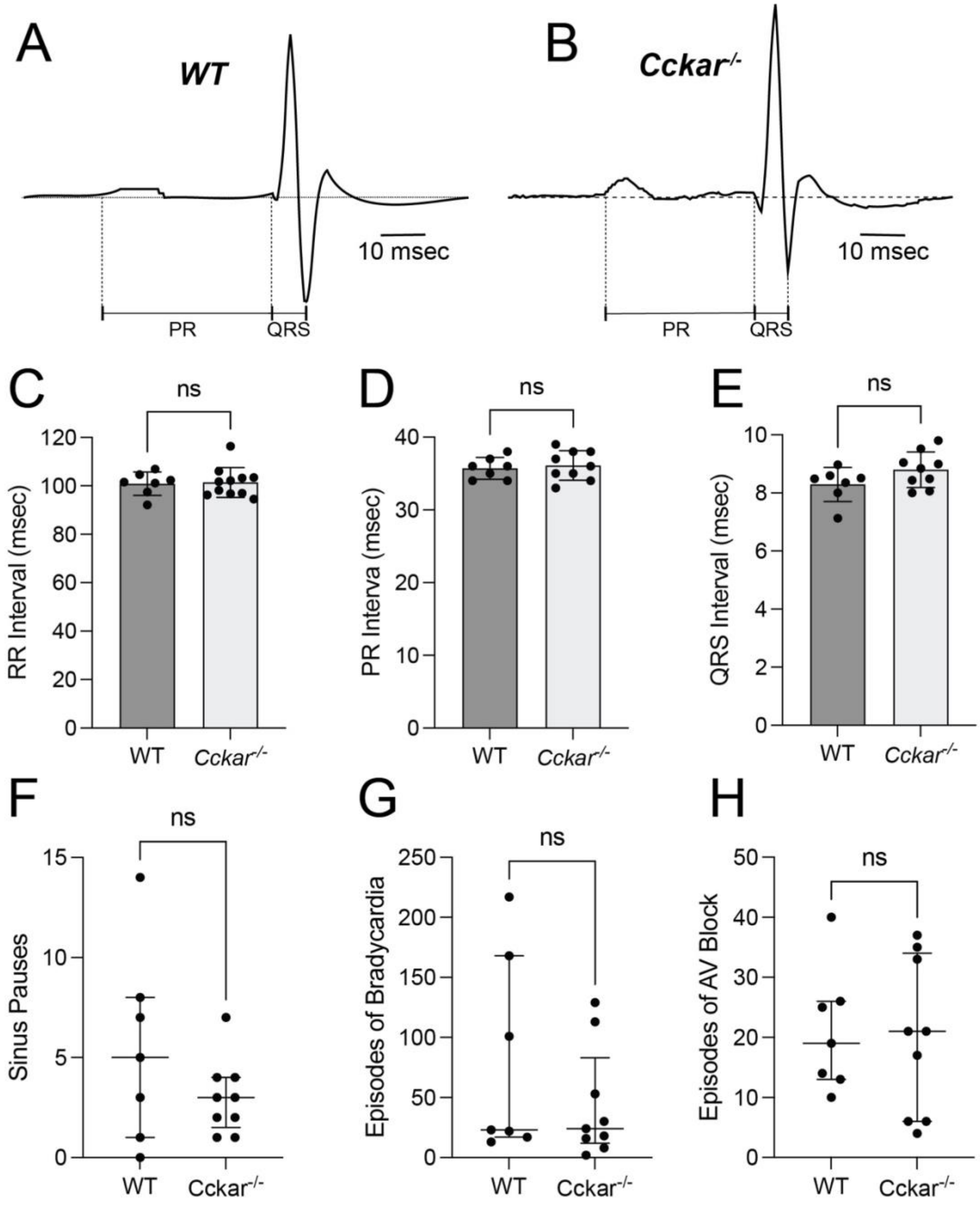

Supplementary Figure 2. Electrocardiographic Intervals and Arrhythmias in WT and *Cckar*$^{-/-}$ Mice. Averaged electrocardiogram (ECG) tracings from representative (A) WT and (B) *Cckar*$^{-/-}$ mice with ECG intervals indicated. (C) Quantification of RR, (D) PR, and (E) QRS intervals from WT (n = 7) and *Cckar*$^{-/-}$ (n = 9) mice. Sinus pauses (F), episodes of sinus bradycardia (G), and episodes of AV block (H) were quantified and summed for each mouse over 48 hours of continuous recording for WT (n = 7) and *Cckar*$^{-/-}$ (n = 9) mice. For electrocardiographic intervals, the line indicates the mean and errors bars indicate standard deviation; for arrhythmia counts, the line indicates the median and the error bars indicate interquartile intervals. For both intervals and arrhythmia episodes, Mann-Whitney tests were used to assess significance ('ns' denotes not significant, '*' denotes $P < 0.5$).

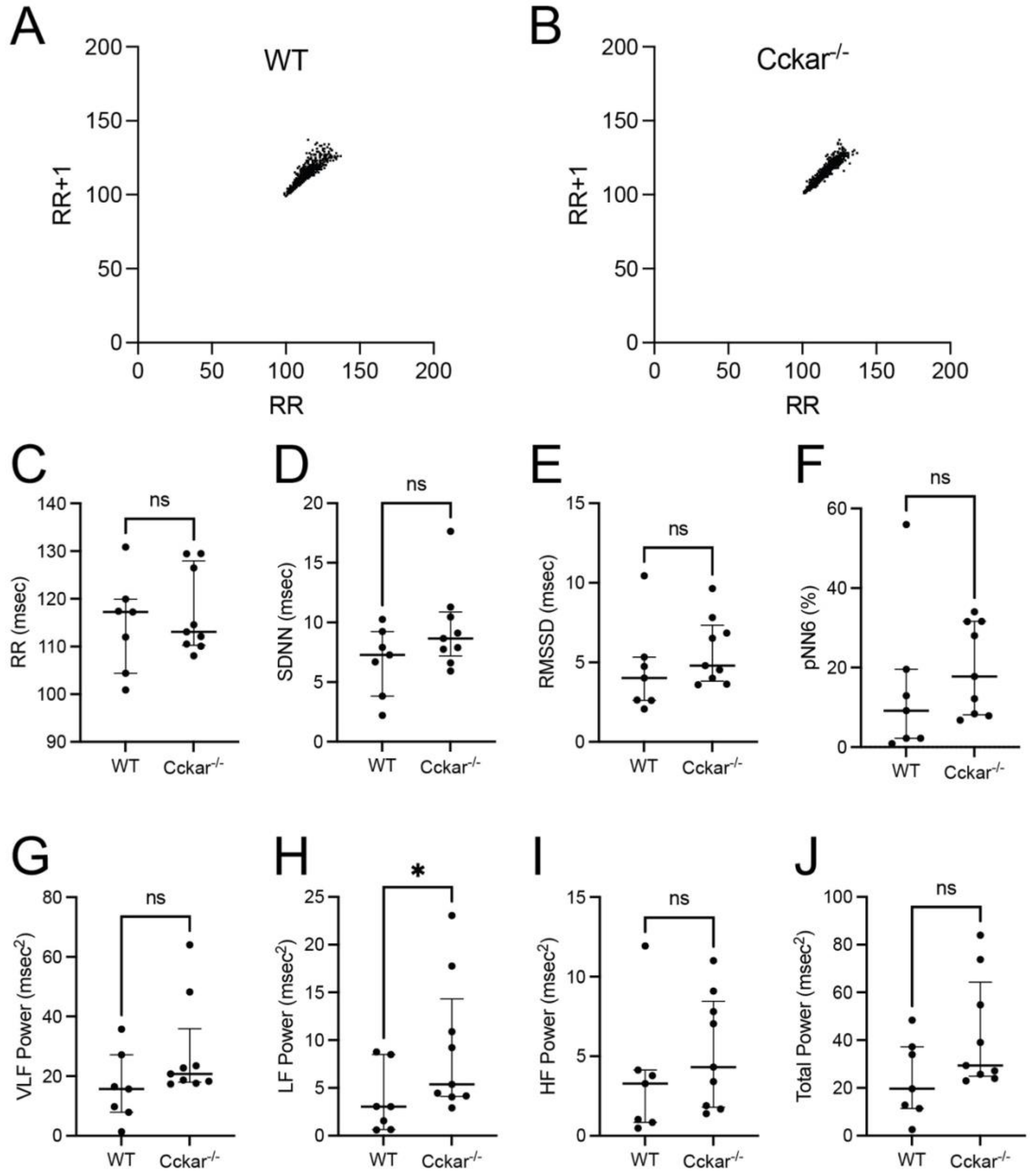

Supplementary Figure 3. Heart Rate Variability in WT and *Cckar*$^{-/-}$ Mice. Poincare plots are shown for a representative WT (A) and (B) *Cckar*$^{-/-}$ littermate during low activity conditions. The following parameters were determined from EKG tracings acquired during normal rhythm from implanted telemetry devices in freely moving unanesthetized WT (n = 7) and *Cckar*$^{-/-}$ (n = 9) littermates: (C) RR interval (D) standard deviation of the RR interval (SDNN), (E) Root mean square of successive differences between heartbeats (RMSSD), and (F) the percentage of successive RR intervals that differ by more than 6 msec (pNN6). Frequency domain analysis was used to calculate the contributions of (G) very low frequency (VLF), (H) low frequency (LF), (I) high frequency (HF) components to (J) the total power. The Mann-Whitney test was used for statistical comparison for all parameters and 'ns' denotes not significant.

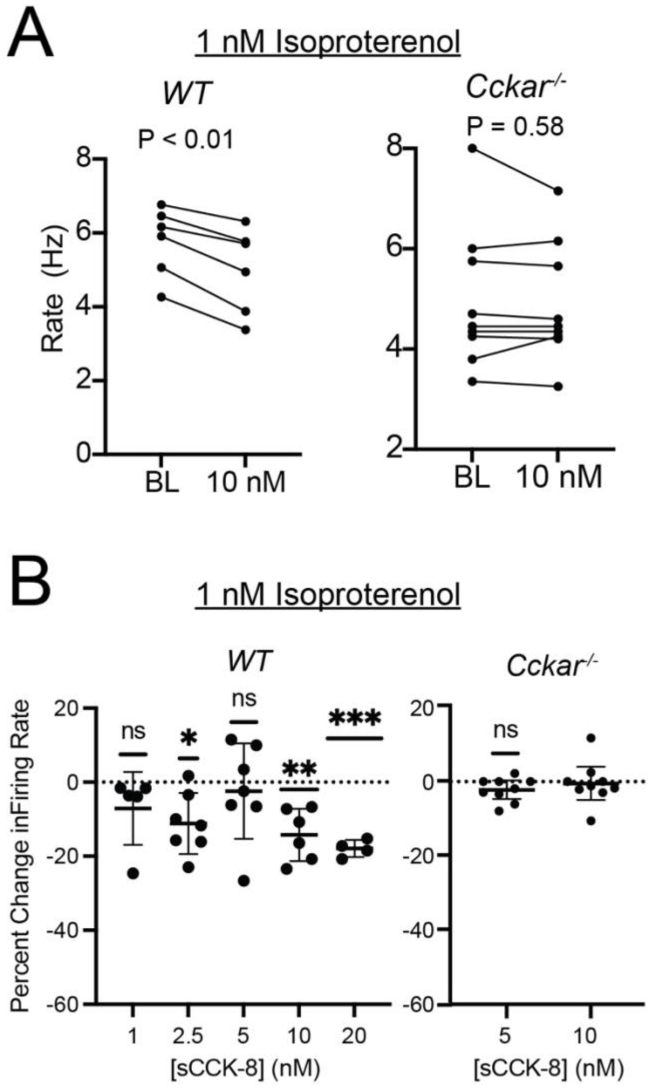

Supplementary Figure 4. Effect of sCCK-8 on Pacemaker Cardiomyocyte Firing Rate in the Presence of Isoproterenol. (A). Reduction in firing rate in response to 10 nM sCCK-8 in the presence of 1 nM isoproterenol for WT (left) and CCKAR$^{-/-}$ (right) pacemaker cardiomyocytes. (B) (left) Percent change in spontaneous firing rate for individual adult WT pacemaker cells 1 minute after perfusion of 1 nM (n=5), 2.5 nM (n=7), 5 nM (n=7), 10 nM (n=6), or 20 nM (n=4) sCCK-8. Statistical comparison between each group and 0% change (null hypothesis) was made with a one sample t-test ('ns' denotes not significant, '*" denotes P < 0.05, '**' denotes P < 0.01, and '***' denotes P < 0.001) (right) Percent change in firing rate in single adult *Cckar*$^{-/-}$ pacemaker cells before and after perfusion with 5 nM (n=9) or 10 nM (n=9) sCCK-8. Statistical comparison between each group and 0% change (null hypothesis) was made with a one sample t-test. Error bars indicate standard deviation.

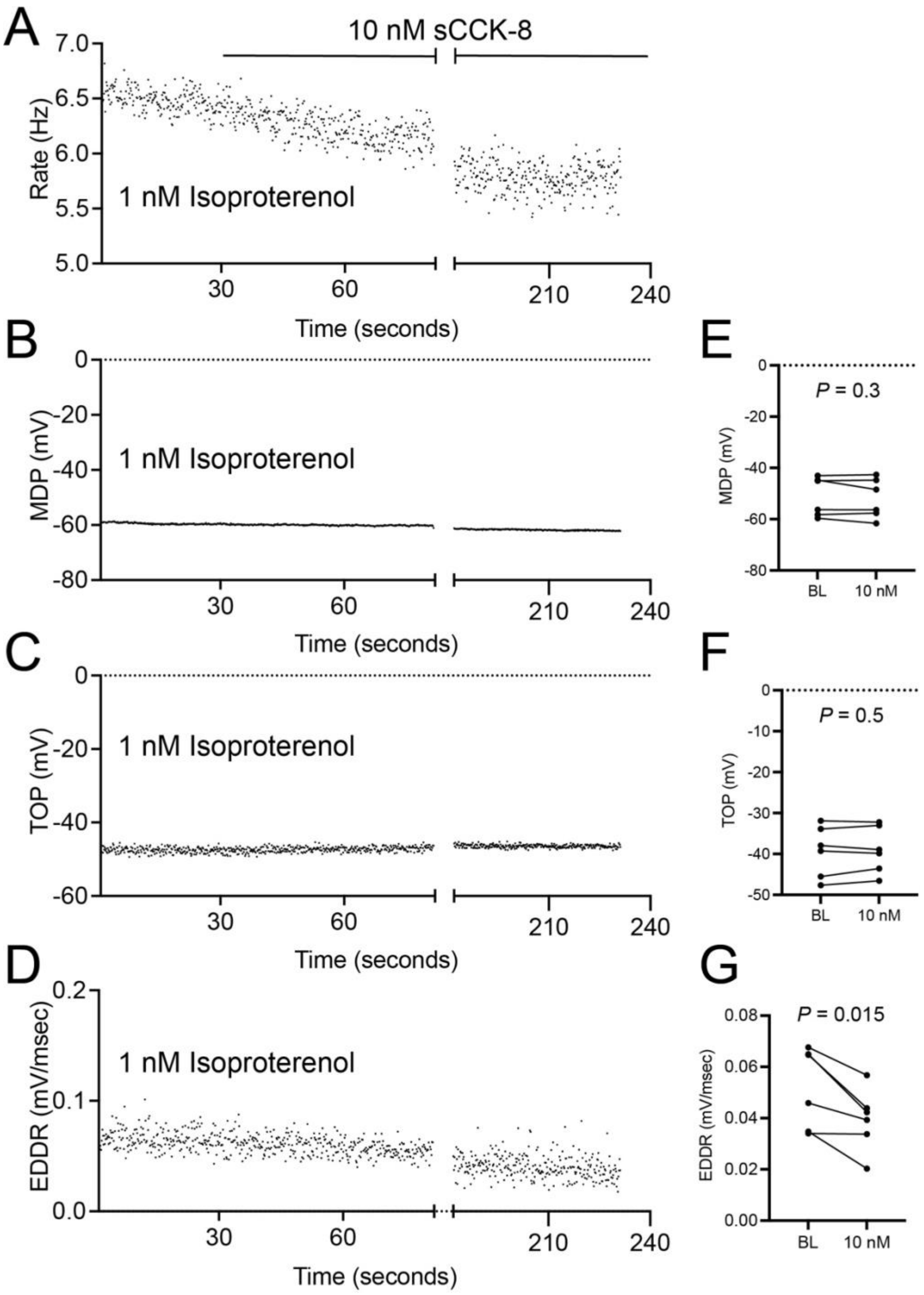

<u>Supplementary Figure 5. Effect of sCCK-8 on Pacemaker Cardiomyocyte Action Potential in the Presence of Isoproterenol.</u> (A). Beat-to-beat change in rate of a single pacemaker cardiomyocyte exposed to 10 nM sCCK-8 in the presence of 1 nM isoproterenol. Shown below are beat-to-beat changes in (B) minimum diastolic potential (MDP), (C) takeoff potential (TOP), and (D) early diastolic depolarization rate (EDDR) rate for the same cell. Shown on the right are before and after comparisons of (E) MDP, (F) TOP, and (G) EDDR for 5 different PCs. P value was calculated with a paired Mann-Whitney test.

Supplementary Table. Differential Expression Analysis for GPCRs: Pacemaker Cardiomyocytes (PC) vs Right Atrial Cardiomyocytes (RA)

ensembl_gene_id	Gene	mRNA Expression (Transcripts per Million)						zScore						Avg z (PC)	P value	Rank	FDR
		RA 1	RA 2	RA 3	PC 1	PC 2	PC 3	RA 1	RA 2	RA 3	PC 1	PC 2	PC 3				
ENSMUSG00000029778	Adcyap1r1	0.61	0.48	1.36	15.67	13.50	16.49	-0.93	-0.95	-0.84	0.96	0.69	1.07	0.91	1.03E-04	2	0.0036
ENSMUSG00000067586	S1pr3	5.40	8.26	11.97	68.39	81.43	87.26	-0.98	-0.91	-0.81	0.63	0.96	1.11	0.90	2.80E-04	4	0.0049
ENSMUSG00000044067	Gpr22	6.04	4.37	3.21	20.10	24.50	23.57	-0.75	-0.92	-1.03	0.64	1.08	0.98	0.90	3.17E-04	5	0.0044
ENSMUSG00000041075	Fzd7	9.43	11.04	8.48	40.12	33.81	44.50	-0.91	-0.81	-0.96	0.93	0.55	1.19	0.89	7.31E-04	6	0.0083
ENSMUSG00000026525	Opn3	3.82	4.08	2.18	10.63	9.39	11.50	-0.77	-0.71	-1.18	0.92	0.61	1.13	0.89	1.12E-03	8	0.0094
ENSMUSG00000037014	Sstr4	0.11	0.39	0.00	17.54	19.93	27.52	-0.88	-0.86	-0.89	0.54	0.74	1.36	0.88	2.03E-03	9	0.0149
ENSMUSG00000029193	Cckar	0.00	0.00	0.05	26.49	37.85	24.04	-0.88	-0.88	-0.87	0.70	1.38	0.55	0.88	2.29E-03	10	0.0149
ENSMUSG00000048216	Gpr85	0.15	0.24	0.23	2.85	3.50	4.59	-0.90	-0.86	-0.86	0.47	0.80	1.35	0.88	2.49E-03	11	0.0145
ENSMUSG00000042429	Adora1	4.38	4.43	2.26	15.32	20.98	14.50	-0.78	-0.77	-1.06	0.66	1.40	0.55	0.87	3.59E-03	12	0.0189
ENSMUSG00000047875	Gpr157	1.84	1.99	1.74	3.28	3.99	3.03	-0.87	-0.71	-0.98	0.69	1.46	0.42	0.85	5.97E-03	13	0.0285
ENSMUSG00000025127	Gcgr	0.95	3.30	1.41	35.58	59.08	68.85	-0.88	-0.81	-0.87	0.24	1.00	1.32	0.85	6.02E-03	14	0.0262
ENSMUSG00000034881	Tbxa2r	0.32	0.79	1.25	7.29	6.89	11.73	-0.95	-0.85	-0.75	0.56	0.47	1.52	0.85	7.57E-03	16	0.0284
ENSMUSG00000028782	Adgrb2	2.83	2.49	1.58	7.67	11.42	6.90	-0.69	-0.78	-1.02	0.57	1.55	0.37	0.83	1.16E-02	18	0.0382
ENSMUSG00000050541	Adra1b	20.68	19.38	16.01	23.17	27.79	32.09	-0.43	-0.65	-1.22	0.00	0.78	1.52	0.77	3.72E-02	24	0.0899
ENSMUSG00000044674	Fzd1	23.63	20.09	19.83	44.36	28.77	32.88	-0.49	-0.87	-0.90	1.72	0.05	0.49	0.75	4.26E-02	26	0.0934
ENSMUSG00000036832	Lpar3	2.21	1.72	2.56	7.26	4.84	12.10	-0.73	-0.85	-0.64	0.54	-0.07	1.75	0.74	5.13E-02	31	0.0928
ENSMUSG00000028036	Ptgfr	2.04	2.37	1.27	2.64	7.57	6.43	-0.64	-0.52	-0.94	-0.41	1.48	1.04	0.70	7.47E-02	35	0.1174
ENSMUSG00000072875	Gpr27	3.12	1.78	3.06	6.05	3.42	7.63	-0.48	-1.09	-0.51	0.85	-0.34	1.57	0.69	7.97E-02	36	0.1196
ENSMUSG00000044017	Adgrd1	1.01	1.96	0.49	6.38	11.28	2.91	-0.73	-0.50	-0.85	0.58	1.76	-0.26	0.69	8.16E-02	37	0.1169
ENSMUSG00000056679	Gpr173	2.40	2.12	2.45	4.41	3.05	6.53	-0.64	-0.81	-0.62	0.54	-0.26	1.79	0.69	8.28E-02	38	0.1133
ENSMUSG00000068740	Celsr2	3.42	2.21	1.24	3.69	3.04	3.99	0.47	-0.69	-1.64	0.74	0.10	1.03	0.62	1.37E-01	40	0.1741
ENSMUSG00000035283	Adrb1	15.90	17.71	10.81	19.09	47.15	22.44	-0.49	-0.35	-0.89	-0.24	1.95	0.02	0.58	1.80E-01	45	0.1999
ENSMUSG00000042793	Lgr6	19.13	26.59	20.16	23.69	40.38	27.58	-0.93	0.04	-0.79	-0.33	1.84	0.17	0.56	1.97E-01	47	0.2055
ENSMUSG00000003476	Crhr2	5.84	6.54	5.47	5.29	7.21	10.99	-0.49	-0.16	-0.67	-0.75	0.15	1.92	0.44	3.31E-01	53	0.2997
ENSMUSG00000022122	Ednrb	10.09	7.39	9.59	9.96	10.71	9.23	0.52	-1.84	0.08	0.41	1.06	-0.24	0.41	3.71E-01	54	0.3229

ensembl_gene_id	Gene	mRNA Expression (Transcripts per Million)						zScore						Avg z (PC)	P value	Rank	FDR
		RA 1	RA 2	RA 3	PC 1	PC 2	PC 3	RA 1	RA 2	RA 3	PC 1	PC 2	PC 3				
ENSMUSG00000031486	Adgra2	7.70	7.38	10.98	10.91	11.43	7.92	-0.89	-1.05	0.84	0.80	1.08	-0.77	0.37	4.28E-01	56	0.3517
ENSMUSG00000034009	Rxfp1	35.59	39.31	23.78	28.85	43.41	42.60	0.00	0.47	-1.50	-0.86	0.99	0.89	0.34	4.63E-01	57	0.3654
ENSMUSG00000013033	Adgrl1	21.05	22.93	18.46	22.25	34.67	17.55	-0.29	0.02	-0.71	-0.09	1.92	-0.85	0.32	4.89E-01	58	0.3708
ENSMUSG00000032492	Pth1r	4.52	5.53	9.15	5.37	9.78	8.11	-1.16	-0.70	0.94	-0.77	1.22	0.47	0.31	5.16E-01	60	0.3700
ENSMUSG00000022297	Fzd6	7.68	5.75	4.50	4.28	7.59	8.26	0.77	-0.34	-1.06	-1.19	0.72	1.11	0.21	6.59E-01	63	0.4390
ENSMUSG00000029090	Adgra3	8.95	9.06	5.80	8.53	8.97	7.56	0.63	0.71	-1.84	0.30	0.65	-0.46	0.16	7.32E-01	65	0.4620
ENSMUSG00000045005	Fzd5	3.80	6.03	4.43	4.23	5.99	4.92	-1.18	1.21	-0.50	-0.72	1.17	0.02	0.16	7.44E-01	66	0.4510
ENSMUSG00000031616	Ednra	89.43	76.86	41.64	79.98	79.66	62.64	1.04	0.30	-1.76	0.48	0.47	-0.53	0.14	7.71E-01	68	0.4424
ENSMUSG00000044197	Gpr146	12.78	11.03	10.11	8.55	16.02	10.64	0.49	-0.19	-0.54	-1.14	1.73	-0.34	0.08	8.65E-01	70	0.4694
ENSMUSG00000042804	Gpr153	8.63	13.96	12.77	8.87	9.24	16.42	-0.94	0.72	0.35	-0.86	-0.75	1.48	-0.04	9.31E-01	71	0.4850
ENSMUSG00000007989	Fzd3	10.04	10.95	9.26	8.82	11.66	9.12	0.06	0.86	-0.63	-1.03	1.49	-0.76	-0.10	8.42E-01	69	0.4393
ENSMUSG00000049791	Fzd4	7.45	7.04	14.08	8.73	8.88	8.82	-0.68	-0.84	1.94	-0.17	-0.11	-0.14	-0.14	7.71E-01	67	0.4027
ENSMUSG00000044337	Ackr3	4.13	3.48	4.45	1.49	5.72	3.05	0.29	-0.17	0.51	-1.56	1.40	-0.47	-0.21	6.60E-01	64	0.3506
ENSMUSG00000118401	Gpr52	8.45	10.72	3.11	3.16	8.60	5.35	0.60	1.31	-1.09	-1.08	0.64	-0.38	-0.27	5.66E-01	62	0.3013
ENSMUSG00000021799	Opn4	10.71	8.57	8.25	5.64	8.17	10.39	1.14	-0.03	-0.20	-1.63	-0.25	0.97	-0.30	5.20E-01	61	0.2727
ENSMUSG00000024958	Gpr137	4.72	4.12	3.98	3.32	4.24	4.35	1.27	-0.01	-0.30	-1.72	0.26	0.50	-0.32	4.93E-01	59	0.2589
ENSMUSG00000036904	Fzd8	3.90	5.32	4.62	3.60	5.20	2.76	-0.33	1.09	0.39	-0.64	0.97	-1.48	-0.38	4.09E-01	55	0.2231
ENSMUSG00000001761	Smo	12.83	17.56	13.41	7.71	14.56	11.83	-0.05	1.41	0.13	-1.62	0.48	-0.35	-0.50	2.63E-01	52	0.1468
ENSMUSG00000000194	Gpr107	7.43	8.64	7.16	5.71	7.85	6.65	0.19	1.39	-0.08	-1.52	0.61	-0.59	-0.50	2.61E-01	51	0.1433
ENSMUSG00000038668	Lpar1	2.21	3.96	11.38	1.44	1.53	2.95	-0.45	0.01	1.98	-0.65	-0.63	-0.26	-0.51	2.45E-01	50	0.1324
ENSMUSG00000044338	Aplnr	2.19	3.11	5.01	2.04	0.06	3.04	-0.24	0.33	1.50	-0.33	-1.55	0.29	-0.53	2.27E-01	49	0.1204
ENSMUSG00000032641	Gpr19	2.66	4.41	3.01	2.18	2.58	2.83	-0.37	1.90	0.09	-0.99	-0.48	-0.15	-0.54	2.15E-01	48	0.1118
ENSMUSG00000045613	Chrm2	87.19	84.50	65.91	64.21	69.21	70.78	1.39	1.11	-0.79	-0.96	-0.45	-0.29	-0.57	1.86E-01	46	0.0970
ENSMUSG00000002871	Tpra1	8.24	7.72	6.68	5.16	5.67	7.40	1.18	0.76	-0.11	-1.37	-0.95	0.49	-0.61	1.46E-01	44	0.0763
ENSMUSG00000024462	Gabbr1	4.73	6.29	4.97	2.71	5.24	2.27	0.23	1.24	0.39	-1.06	0.56	-1.35	-0.62	1.39E-01	43	0.0712
ENSMUSG00000030406	Gipr	31.30	16.10	4.12	4.56	2.04	0.69	1.81	0.53	-0.48	-0.44	-0.65	-0.77	-0.62	1.37E-01	42	0.0685
ENSMUSG00000028184	Adgrl2	19.91	21.42	15.25	12.65	17.49	12.86	0.91	1.32	-0.37	-1.08	0.25	-1.03	-0.62	1.37E-01	41	0.0668
ENSMUSG00000059588	Calcrl	5.38	5.17	5.56	1.95	3.68	5.07	0.65	0.50	0.78	-1.79	-0.56	0.43	-0.64	1.18E-01	39	0.0577

ensembl_gene_id	Gene	mRNA Expression (Transcripts per Million)						zScore						Avg z (PC)	P value	Rank	FDR
		RA 1	RA 2	RA 3	PC 1	PC 2	PC 3	RA 1	RA 2	RA 3	PC 1	PC 2	PC 3				
ENSMUSG00000036353	P2ry12	1.16	6.35	6.36	0.64	0.17	0.30	-0.44	1.28	1.28	-0.62	-0.77	-0.73	-0.71	7.07E-02	34	0.0374
ENSMUSG00000045092	S1pr1	21.54	17.87	21.06	14.37	15.67	18.25	1.20	-0.09	1.03	-1.32	-0.86	0.04	-0.71	6.66E-02	33	0.0343
ENSMUSG00000039942	Ptger4	2.22	4.89	7.58	0.63	0.21	0.97	-0.18	0.74	1.66	-0.73	-0.87	-0.61	-0.74	5.14E-02	32	0.0257
ENSMUSG00000052336	Cx3cr1	2.14	9.65	9.70	0.22	0.19	0.21	-0.33	1.27	1.28	-0.74	-0.74	-0.74	-0.74	5.04E-02	30	0.0252
ENSMUSG00000050199	Lgr4	26.92	27.19	19.89	16.30	19.50	16.42	1.20	1.26	-0.23	-0.97	-0.31	-0.94	-0.74	4.97E-02	29	0.0240
ENSMUSG00000049130	C5ar1	7.80	17.44	29.14	0.78	0.59	1.24	-0.15	0.68	1.69	-0.75	-0.77	-0.71	-0.74	4.90E-02	28	0.0227
ENSMUSG00000020591	Ntsr2	5.03	4.61	3.89	1.83	3.55	2.95	1.20	0.84	0.21	-1.56	-0.08	-0.60	-0.75	4.54E-02	27	0.0202
ENSMUSG00000021886	Gpr65	1.28	4.44	4.83	0.00	0.00	0.17	-0.22	1.17	1.35	-0.79	-0.79	-0.72	-0.77	3.72E-02	25	0.0164
ENSMUSG00000008734	Gprc5b	4.40	6.63	8.12	2.77	3.11	3.17	-0.14	0.88	1.56	-0.88	-0.72	-0.70	-0.77	3.65E-02	23	0.0159
ENSMUSG00000040552	C3ar1	8.81	17.87	27.40	0.72	0.60	1.36	-0.06	0.76	1.62	-0.79	-0.80	-0.73	-0.77	3.32E-02	22	0.0136
ENSMUSG00000002885	Adgre5	18.87	27.26	25.36	6.62	6.84	14.75	0.25	1.20	0.99	-1.13	-1.10	-0.21	-0.81	1.74E-02	21	0.0066
ENSMUSG00000048779	P2ry6	3.23	6.89	7.70	0.22	0.04	0.89	0.02	1.09	1.33	-0.86	-0.92	-0.67	-0.82	1.65E-02	20	0.0058
ENSMUSG00000043953	Ccrl2	21.36	25.65	33.57	11.78	12.62	13.16	0.19	0.68	1.58	-0.90	-0.80	-0.74	-0.82	1.63E-02	19	0.0052
ENSMUSG00000033446	Lpar6	4.06	4.60	3.81	2.49	3.09	2.40	0.73	1.34	0.45	-1.03	-0.36	-1.13	-0.84	9.18E-03	17	0.0027
ENSMUSG00000058396	Gpr182	4.76	3.88	3.11	1.38	0.35	1.11	1.34	0.83	0.39	-0.61	-1.19	-0.76	-0.85	6.26E-03	15	0.0017
ENSMUSG00000050288	Fzd2	7.89	6.74	7.78	4.17	4.26	4.15	1.12	0.49	1.06	-0.90	-0.86	-0.91	-0.89	8.97E-04	7	0.0004
ENSMUSG00000036437	Npy1r	28.31	23.18	24.82	3.55	2.68	3.91	1.14	0.72	0.85	-0.89	-0.96	-0.86	-0.90	1.44E-04	3	0.0001
ENSMUSG00000048376	F2r	58.44	52.68	59.36	18.10	14.47	15.12	0.98	0.72	1.02	-0.81	-0.97	-0.94	-0.91	6.59E-05	1	0.0001

SUPPLEMENTARY METHODS

Animal Handling and Maintenance

Mouse studies were performed in accordance with IACUC-approved protocols at the University of California, San Francisco. Mouse lines were maintained on C57Bl6. *Cckar*^{tm1Kpn} (hereafter, *Cckar*$^{-/-}$) mice were generated by Alan Kopin et al. and were obtained from Nirao Shah's lab [2]. *Cck*$^{tm1.1(cre)Zjh}$ (hereafter, *Cck*^{Cre}) mice were obtained from Jackson Labs. *Gt(ROSA)26Sor*$^{tm4(ACTB\text{-}tdTomato,\text{-}EGFP)Luo}$ (hereafter, *ROSA*$^{mT/mG}$ mice were generated by Muzumdar et al. and were obtained from Brian Black's lab [3].

Analysis of Bulk RNA Sequencing Data

To identify G-protein coupled receptors (GPCRs) that were expressed in pacemaker cardiomyocytes (PCs) and right atrial cardiomyocytes (RACMs), respectively, RNA-seq data from neonatal sorted mouse PCs and RACMs were downloaded, and the list of genes expressed in each tissue as defined by average transcripts per million across 3 biological replicates greater than 3 was intersected with an annotated database of from the International Union of Basic and Clinical Pharmacology. For each GPCR identified, a false discovery rate was calculated for differential expression was determined using the method of Benjamini-Hochberg.

Single Cell RNA Sequencing Analysis

Mouse E16.5 SAN s 10x Genomics Mouse single-cell RNA-sequencing files were generated by Goodyer et al. [4]and downloaded from GEO (GSE132658). Adult rabbit single-cell RNA-seq data were generated by Liang et al.[1] and were downloaded as fastq files from the NCBI Sequence Read Archive (PRJNA531288). Fastq files from the rabbit dataset were trimmed using Fastp[5] and aligned to the oryCun2 assembly (UCSC genome browser) using Hisat2[6]. Finally, an expression matrix was constructed for each cell using featureCounts[7]. Both mouse and rabbit datasets were filtered, clustered, and analyzed using Scanpy[8] to identify the cell types expressing both *Cckar* and *Cck*.

Quantitative PCR

The SAN and RA regions from 3 adult hearts were manually dissected using anatomical landmarks to delineate the inter-caval region. For each sample, total RNA from SAN and right atrium tissue was extracted using TRIzol reagent (Life technologies, CA, USA) and RNeasy micro kit (QIAGEN) according to the manufacturer's instructions. 0.2 micrograms of total RNA were used to synthesize the first-strand cDNA with Superscript III first-strand synthesis kit (Invitrogen). Real time qPCR was performed using Taqman fast advanced master mix (Thermo Fisher Scientific, USA). GAPDH was used

as an endogenous control with the $\Delta\Delta$CT method for comparison of SAN and right atrial tissue. A Mann-Whitney test was used for significance with $P < 0.05$ deemed significant.

Immunohistochemistry

Freshly isolated hearts were perfused with PBS, fixed in 4% PFA overnight at 4°C, washed in PBS, incubated in 15% sucrose followed by 30% sucrose, and then embedded in Optimal Temperature Cutting medium (Tissue Tek). 7.0 µm sections were cut with a Leica CM3050S cryostat. For antibody staining, sections were blocked in 0.1% saponin and 1% casein solution (Thermo Fisher, Catalog#37582) for 1 hour at room temperature and incubated with primary antibody overnight at 4°C (rabbit anti-Hcn4, Alomone Labs, APC-052, 1:300; chicken anti-GFP, Aves Labs GFP-1010, 1:300; goat anti-TdTomato, Origene, AB8181-200, 1:400). Sections were incubated with appropriate AlexaFluor conjugated secondary antibodies at 1:1000 for 1 hour at room temperature. DAPI was used to visualize the nuclei.

For acetylcholinesterase staining, sections were briefly washed with PBS, incubated in the reaction solution, which contains (in 10 ml of volume: acetyl thiocholine iodide 5mg; 0.1M sodium acetate (pH 6.0) 6.5ml; 0.1M sodium citrate 0.5ml; 30mM copper sulphate 1.0ml; 5mM potassium ferricyanide 1.0ml) for 2 hours at room temperature, rinsed with water and mounted.

Transmitter Implantation and Analysis of Heart Rhythm Data

Adult mice (8-12 weeks old) underwent subcutaneous implantation of telemetry devices (ETA-F10, Data Science International, MN) under anesthesia according to the manufacturer’s instructions. After at least 7 days of recovery, continuous ECG signals were sampled using Ponemah software (DSI) at 1.0 kHz on freely moving and conscious mice for 48 hours per mouse. Maximum heart rate was ascertained after injection of 1.0 µg of isoproterenol. To determine the intrinsic heart rate, a separate cohort of mice were injected with 3.0 mg/kg atropine and 10.0 mg/kg propranolol intraperitoneally under anesthesia with 1% isoflurane. Anesthetized ECG signals were recorded with a Power Lab and Animal BioAmp (AD Instruments) and analyzed offline using ChartPro (AD Instruments).

Heart rhythm analysis on data from implanted transmitters was performed offline using Ponemah (Data Sciences International, St Paul, MN). 48 hours of heart rhythm data were analyzed for each mouse, with determination of average heart rate and PR interval. To generate heart rate histograms, two 12-hour segments corresponding to low activity (8AM-8PM) and two 12-hour segments corresponding to high activity (8PM-8AM) were analyzed for each mouse. The average heart rate for each minute over the 48-hour period was sorted into 10-bpm bins, with the data from low and high activity phases plotted and analyzed separately. To measure the intrinsic heart rate, a 5-minute period of stable heart rate after injection of atropine and propranolol was averaged for each mouse tested. Similarly, maximum heart was

determined by averaging over 1 minute of continuous and stable heart rhythm after injection of 1.0 μg isoproterenol.

For identification of arrhythmias, RR intervals that exceeded twice the RR average of the low- or high-activity period in which they occurred were identified automatically using Ponemah. If an RR interval was longer than twice the average of the previous three beats, it was classified as a sinus pause or, if a P wave was present without a QRS complex, an episode of AV block. Flagged RR intervals that did not meet the criteria for a pause but that exceeded twice the average RR were classified as bradycardic episodes. Low activity and high activity periods for each mouse had separate RR cutoffs determined by the average RR interval from 8AM-8PM (low activity) and 8PM-8AM (high activity). Animals of each genotype were grouped and between group statistical comparisons were made with the Mann-Whitney test with $p < 0.05$ was deemed significant.

Heart rate variability analysis was performed using Ponemah and MATLAB as previously described with minor modifications [9]. Low and high activity periods were analyzed separately. Poincare plots for a representative WT and *Cckar*$^{-/-}$ mouse were created using 2 minutes of consecutive heart beats acquired during an interval of stable rhythm in a low activity period. For time-domain analysis, three 10-minute segments were selected from a 2-hour time window for each mouse (12PM -2PM for low activity period and 12AM – 2AM for high activity period). Outliers greater than 2 standard deviations from the mean were removed. The average NN interval, standard deviation of all RR intervals (SDNN), root mean square of the difference of successive RR intervals (RMSSD), and percentage of normal consecutive RR intervals that differ by more than 6 msec (pNN6) were calculated in Ponemah. Statistical comparisons were made with the Mann-Whitney test and $p < 0.05$ was deemed significant.

Isolation of Adult Mouse Cardiac Pacemaker Cells

Excised hearts were placed in normal Tyrode's solution, which contained (in mmol/L): 140 NaCl, 5.4KCl, 1.0 $CaCl_2$, 1.2 KH_2PO_4, 5.0 HEPES, 1.0 MaCl2, 5.55 glucose, pH7.4 with NaOH. The SAN was isolated by excising the region of the right atrium bounded by the superior vena cava, inferior vena cava, interatrial septum, and crista terminalis. Isolated SAN tissue was cut into 1 mm wide strips and placed in the Ca2+ free Tyrode's solution with liberase (0.18 mg/ml, Roche, CA, USA) and elastase (2.0 U/ml) at 37 degree for 25 min. The SAN tissue was further dissociated to a single cell suspension by trituration with a glass pipette in KB solution, which contained (in mmol/L): 25 KCl, 10 KH_2PO_4, 5 HEPES, 20 Glucose, 20 taurine, 100 K-glutamate, 10 K-aspartate, 2.0 Mg_2SO_4, 5.0 Creatine, 0.5 EGTA, pH7.2 with KOH. The cell suspension was stored at 4°C, and re-equilibrated for at least 30 minutes at room temperature immediately before electrophysiological recording.

Whole Cell Patch Clamp

Action potentials were acquired from spontaneously beating adult cells of the indicated genotypes via the perforated patch-clamp technique with an Axopatch-700B amplifier at 32°C or 35°C using pCLAMP10.3 (Molecular Devices/Axon Instruments, Sunnyvale, CA) for data acquisition. All recordings were digitized at 20 KHz and filtered at 1.0 kHz. Electrodes had resistances of 3.5-4.0 MΩ. The bath solution was normal Tyrode's solution. The pipette solution contained (in mmol/L): 130 KCl, 10 NaCl, 0.04$CaCl_2$, 2 Mg_2ATP, 7 phosphocreatine, 0.1NaGTP, 10 HEPES and 200ug/ml of Amphotericin B (pH 7.2 with KOH). The pCLAMP 10.6 Clampfit module was used for analysis of action potential parameters. Firing rate was averaged from a 10 sec gap-free interval of stable recording. The change in firing rate was continuously recorded before and after the application of sCCK-8 (TOCRIS, cat# 1166) diluted in Tyrode solution at indicated concentrations.

The pCLAMP 10.6 Clampfit module and LabChart7 were used for analysis of action potentials (APs). To obtain the early diastolic depolarization velocity (EDDR), a take-off potential (TOP) was defined as the membrane potential when the dV/dT reached 10% of the maximum of the first derivative of the AP tracing (dV/dt_{max}). The total diastolic duration was defined as the time between the minimum diastolic potential (MDP) and TOP. Early diastolic depolarization (EDD) was defined as the time between 10% and 50% of the total diastolic duration. The EDDR was then defined as the change in membrane voltage in early diastole divided by the duration of EDD. Statistical comparisons of AP parameters before and after application of s-CCK8 were made with paired t-tests after each distribution passed a test of normality. $P < 0.05$ was deemed statistically significant.

REFERENCES

1. Liang D, Xue J, Geng L, Zhou L, Lv B, Zeng Q, Xiong K, Zhou H, Xie D, Zhang F, Liu J, Liu Y, Li L, Yang J, Xue Z, Chen YH. Cellular and molecular landscape of mammalian sinoatrial node revealed by single-cell rna sequencing. *Nat Commun*. 2021;12:287

2. Kopin AS, Mathes WF, McBride EW, Nguyen M, Al-Haider W, Schmitz F, Bonner-Weir S, Kanarek R, Beinborn M. The cholecystokinin-a receptor mediates inhibition of food intake yet is not essential for the maintenance of body weight. *J Clin Invest*. 1999;103:383-391

3. Muzumdar MD, Tasic B, Miyamichi K, Li L, Luo L. A global double-fluorescent cre reporter mouse. *Genesis*. 2007;45:593-605

4. Goodyer WR, Beyersdorf BM, Paik DT, Tian L, Li G, Buikema JW, Chirikian O, Choi S, Venkatraman S, Adams EL, Tessier-Lavigne M, Wu JC, Wu SM. Transcriptomic profiling of the developing cardiac conduction system at single-cell resolution. *Circulation Research*. 2019;125:379-397

5. Chen S, Zhou Y, Chen Y, Gu J. Fastp: An ultra-fast all-in-one fastq preprocessor. *Bioinformatics*. 2018;34:i884-i890

6. Kim D, Langmead B, Salzberg SL. Hisat: A fast spliced aligner with low memory requirements. *Nat Methods*. 2015;12:357-360

7. Liao Y, Smyth GK, Shi W. Featurecounts: An efficient general purpose program for assigning sequence reads to genomic features. *Bioinformatics*. 2014;30:923-930

8. Wolf FA, Angerer P, Theis FJ. Scanpy: Large-scale single-cell gene expression data analysis. *Genome Biol*. 2018;19:15

9. Fenske S, Probstle R, Auer F, Hassan S, Marks V, Pauza DH, Biel M, Wahl-Schott C. Comprehensive multilevel in vivo and in vitro analysis of heart rate fluctuations in mice by ecg telemetry and electrophysiology. *Nat Protoc*. 2016;11:61-86
